# Heterogeneous correlate and potential diagnostic biomarker of tinnitus based on nonlinear dynamics of resting-state EEG recordings

**DOI:** 10.1371/journal.pone.0290563

**Published:** 2024-01-02

**Authors:** Zahra Naghdabadi, Mehran Jahed

**Affiliations:** Department of Electrical Engineering, Sharif University of Technology, Tehran, Iran; Northeastern University, UNITED STATES

## Abstract

Tinnitus is a heterogeneous condition of hearing a rattling sound when there is no auditory stimulus. This rattling sound is associated with abnormal synchronous oscillations in auditory and non-auditory cortical areas. Since tinnitus is a highly heterogeneous condition with no objective detection criteria, it is necessary to search for indicators that can be compared between and within participants for diagnostic purposes. This study introduces heterogeneous though comparable indicators of tinnitus through investigation of spontaneous fluctuations in resting-state brain dynamics. The proposed approach uses nonlinear measures of chaos theory, to detect tinnitus and cross correlation patterns to reflect many of the previously reported neural correlates of tinnitus. These indicators may serve as effective measures of tinnitus risk even at early ages before any symptom is reported. The approach quantifies differences in oscillatory brain dynamics of tinnitus and normal subjects. It demonstrates that the left temporal areas of subjects with tinnitus exhibit larger lyapunov exponent indicating irregularity of brain dynamics in these regions. More complex dynamics is further recognized in tinnitus cases through entropy. We use this evidence to distinguish tinnitus patients from normal participants. Besides, we illustrate that certain anticorrelation patterns appear in these nonlinear measures across temporal and frontal areas in the brain perhaps corresponding to increased/decreased connectivity in certain brain networks and a shift in the balance of excitation and inhibition in tinnitus. Additionally, the main correlations are lost in tinnitus participants compared to control group suggesting involvement of distinct neural mechanisms in generation and persistence of tinnitus.

## Introduction

Tinnitus is often explained as the perception of sound in the absence of external acoustic stimulus. It is an annoying condition prevalent in more than 10% of adults [1] and a global burden [2]. Sometimes, tinnitus coexists with hyperacusis or depression and occasionally it is followed by hearing loss [1, 3]. There is no certain cure for tinnitus due to the many challenges of investigating this condition [4]. The lack of an objective measure for detecting tinnitus, the heterogeneous nature of tinnitus, and the lack of standard methods for tinnitus research studies has made tinnitus assessment difficult [5, 6]. Tinnitus heterogeneity and the identification of phenotypes is still under investigation [7]. Even existence of a common pathological mechanism for tinnitus emergence is unknown [8].

Tinnitus is associated with malfunctioning of certain networks in the brain. Resting-state studies have revealed abnormal spontaneous activity [9, 10] as well as network alterations in widely distributed brain areas [11] such as ventromedial prefrontal cortex (vmPFC) [12, 13], dorsolateral prefrontal cortex (dlPFC) [14, 15], anterior cingulate cortex (ACC) [15–19], parahippocampus (PHC) [18, 20], amygdala (AMY) [21], insula (INS) [16, 22], and the primary auditory cortex (A1) [23]. Accordingly, tinnitus has been viewed as a complex brain disorder that involves alterations in brain networks mediating perception, distress, salience, memory and attention [24].

Several mechanisms have been proposed as the neural basis of tinnitus including permanent alterations in the ongoing oscillatory dynamics at higher layers of the auditory hierarchical stream [25]. On the other hand, the theory of nonlinear dynamical systems also called “chaos theory” has been useful in the study of human brain in complex conditions such as epilepsy [26, 27], dementia [28] and psychiatric diseases [29]. Such nonlinear time series analysis has been applied to EEG and MEG recordings of healthy participants during perceptual processing, performance of cognitive tasks, different stages of sleep, and no-task states [30].

The method of nonlinear dynamics is also useful for diagnostic purposes in Alzheimer’s disease [31, 32] and its early recognition [33]. It has also been proposed for clinical application in dementia and Parkinson’s disease [34]. Moreover, use of chaos theory has been useful in predicting epileptic seizures [35] and computer modeling of epilepsy [36–38]. In 2001, nonlinear dynamics of standard EEG recordings successfully predicted seizures in 23 patients with temporal lobe epilepsy [39]. Recent advances make it possible for seizure prediction in clinical trials and implantable devices [40]. However, to the best of our knowledge, use of nonlinear measures from chaos theory has not been reported in tinnitus studies and no objective measure of tinnitus is reported in the literature.

In tinnitus, the increased synchrony observed in the firing and bursting rate of neurons in auditory cortex, is considered the fundamental perceptual correlate of phantom sound perception [25, 41]. But it is unknown how this altered oscillatory behavior or its correlates can be quantified and used as biomarkers to detect tinnitus in humans. If the rattling sound in tinnitus is due to abnormal oscillations of neurons, then it affects nonlinear dynamics of the brain as previously reported [10]. In this study, using time-delay embedding of resting-state EEG recordings, we calculate quantitative measures of chaos. In this method, the time-delay used for attractor reconstruction is optimized for each channel-participant and hence is a heterogeneous parameter on which the rest of analysis depends on. We use topological entropy and the largest lyapunov exponent as measures of unpredictability, irregularity, and complexity [42, 43] to quantify and differentiate nonlinear dynamics in temporal and frontal areas of the brain. We demonstrate that these measures separate tinnitus from normal group and support several neural mechanisms previously proposed for tinnitus generation and persistence.

## Materials and methods

### Participants

A total of 50 participants were recruited for a larger EEG study [44]. Resting-state recordings of fourteen participants were included in this study. The study was approved by the Ethics Committee of Iran University of Medical Sciences (IUMS; ENT and Head and Neck Research Center), through Ethics Code of IR.IUMS.REC.1393.9011369004. All participants provided written informed consent to participate in the study. A total of 1106 seven-second data windows (1053 tinnitus and 53 normal) were recorded from subjects. Data from seven participants (4 males and 3 females, age: 50 ± 12) who perceived tinnitus and seven normal individuals as control group (4 males and 3 females, age: 30.3 ± 8.1), were used in this study. All participants with and without tinnitus had normal hearing as well as normal external and middle ear function checked via otoscopy and tympanometry. Participants with at least one of the following criteria were excluded from the study: history of auditory or neurological disorders; tinnitus secondary to a systemic disease; head and neck disease or space-occupying lesion; history of exposure to head trauma, excessive noise, acoustic trauma, or ototoxic agents; use of neurological/psychiatric medications within the past 3 months; receiving treatment for tinnitus within the past 3 months; pregnancy or breastfeeding; temporomandibular disorders; and alcohol or drug abuse.

The patient group were diagnosed with subjective idiopathic tinnitus (>6 months), confirmed by an audiologist. The patient group had behavioral pure tone audiometry (PTA) threshold levels <20dB hearing level (HL) in octave frequencies of 250–2000 Hz and not more than 40dB HL in frequencies of 4–8 kHz (for details about participants see [44]).

### Data acquisition

Participants were told to relax their muscles and minimize eye movements and blinks, while being seated in an acoustically and electrically shielded room. Resting-state EEG was recorded from 29 scalp sites (FP1, FPz, FP2, F7, F3, Fz, F4, F8, FT7, FC3, FCz, FC4, FT8, T7, C3, Cz, C4, T8, TP7, CP3, CPz, CP4, TP8, P3, Pz, P4, POz, M1, and M2) chosen according to the International 10–20 system using 64-channel BRAIN QUICK LTM (Micromed, Italy) referenced to the tip of the nose. The sampling frequency was 1 kHz and an online band-pass filter of 0.4–200 Hz was used during recording. Participants went through an auditory task explained in [44] after open-eyed resting-state recordings.

### Data analyses

Data analyses was done in EEGLAB and MATLAB 2019b, and 2021b. Nonlinear features were calculated using standard built-in functions from Predictive Maintenance Toolbox. Here, we explain utilized algorithms briefly.

#### Preprocessing

We used resting-state EEG recordings of participants. Data was carefully inspected and band pass filtered at 0.5–100 Hz. Then EOG, ECG, and eye blink artifacts were removed using ICA with 29 components (runica algorithm in EEGLAB) [45]. After ICA, data was segmented into windows with 7 seconds in length and 2 seconds of overlap. The window length was kept more than three times the period of the smallest frequency available in the signal.

#### Nonlinear feature extraction

The first step in nonlinear analysis of EEG is reconstructing the attractor and the phase space which is proved to have the same properties as the true attractor of the underlying dynamical system [46]. In this step, we find the optimal lag and embedding dimension for each EEG channel of each participant independently. Therefore, reconstructing the attractor and calculating nonlinear measures from the attractor are performed in a heterogeneous manner.

*Phase space reconstruction*. The phase space was reconstructed using time-delay embedding (MATLAB function *phaseSpaceReconstruction)* for each data window separately. Taking *X* = [*x*(1), *x*(2),…, *x*(*N*)] as a uniformly sampled time series (e.g. one window), the reconstructed phase space with lag *τ* and embedding dimension *m* is generated by points of the form *Y* = [*x*(*t*), *x*(*t* − *τ*), x(*t* − 2*τ*),…, *x*(*t* − (*m* − 1)*τ*)] [43].

*Delay estimation*. The optimal delay (or lag) is estimated as the first local minimum of Average Mutual Information (AMI) between the main time series and its delayed version defined as

$$AMI(\tau )=\sum_{i=1}^{N}p(x(i),x(i+\tau )){\text{log}}_{2}\left[\frac{p(x(i),x(i+\tau ))}{p(x(i))p(x(i+\tau ))}\right]$$

for *τ* between 1 and 10 milliseconds [43, 47].

*Embedding dimension* is estimated using False Nearest Neighbors (FNN) Algorithm [48]. False Nearest Neighbors of a point *Y* in the phase space consists of all points Y* with

$$\sqrt{\frac{R_{i}^{2}(d+1)-R_{i}^{2}(d)}{R_{i}^{2}(d)}}>\text{DistanceThreshold}$$

where $R_{i}^{2}(d)={\left\|Y-\left.Y^{*}\right\|\right.}^{2}$, is the distance metric. The embedding dimension is estimated as the smallest value *d* that satisfies the condition *p*_*fnn*_*< PercentFalseNeighbors*, where *p*_*fnn*_ is the ratio of FNN points in the reconstructed phase space (see [48] for details).

*Approximate entropy* was calculated as Φ_*m*_ − Φ_*m*+1_ with $\Phi_{m}={\left(N-m+1\right)}^{-1}\sum_{i=1}^{N-m+1}\text{log}(N_{i})$ where *N*_*i*_ is the number of points within distance *R* from point *Y* [35].

*Lyapunov exponent* is the rate of separation of infinitesimally close trajectories in the phase space and is used to quantify the level of chaos in a system [49]. It is often calculated by considering a number of nearby points and quantifying the exponential increase or decrease of vector distances during time intervals [30]. Lyapunov exponent in the entire range (*[k*_*min*_ *k*_*max*_*]*) was calculated as

$$\lambda (i)=\frac{1}{(K_{\text{max}}-K_{\text{min}}+1)dt}\sum_{k=K_{\text{min}}}^{k=K_{\text{max}}}\frac{1}{K}\;\text{ln}\;\frac{\left\|Y(i+k)-Y(i*+k)\right\|}{\left\|Y(i)-Y(i*)\right\|}.$$

In the above formula, [*k*_*min*_ *k*_*max*_] is the interval in which *λ*(*i*) is linear, where *k*_*min*_ = *100* and *k*_*max*_ = *300* for windows of length 7 sec. Here Y(i)* is the nearest point to *Y(i)* satisfying |*i* − i*| < MinSeparation with *MinSeparation* being equal to reciprocal of mean frequency [49].

Nonlinear measures were calculated for all data windows using *approximateEntropy* and *lyapunovExponent* functions in the Predictive Maintenance Toolbox in MATLAB.

*Surrogate data testing*. The existence of nonlinear structure in data was confirmed by using phase-shuffled surrogates [50]. In this method, we calculate Fourier transform of the original signal, generate a vector of random phases and take the inverse Fourier transform using random phases. These surrogates preserve linear behavior, the power spectrum/autocorrelation, but destroy any nonlinear structure [51]. We generated surrogates for 1% of data points and their nonlinear measures were calculated after phase space reconstruction using the same lag and embedding dimension as original corresponding data point. Significance of nonlinear measures were investigated using two-sampled t-tests.

*Statistical testing*. To test significance of nonlinear measures between patient and normal groups, we performed two-sampled t-tests for all metrics and the marking convention is *p < 0.05, **p < 0.01, *** p < 0.001 (two-sided).

*Classification*. Nonlinear measures obtained from 25 channels were used as features of trials (n = 1053 in tinnitus and n = 53 in control group). Linear SVM classifiers were trained for this dataset using 5-fold cross validation. Data was split randomly into 5 segments equal in length in each group. We constructed 5 classifiers using different testing and training sets. Train size was 1097 and test size was 274 for each classifier. Hence, for each classifier, one segment of data was employed as the testing set and the other 4 segments were used to train the model. Accuracy, true positive rate, and true negative rate were computed for each classifier. We reported the mean and standard deviation of accuracy, true positive rate, and true negative rate in the classification results.

To compensate for group size differences, data points coming from the control group were considered with the weight of seven. This weight was the smallest number where performance metrics became stationary and did not change with increasing the weight. Classifiers were trained based on different feature sets based on nonlinear measure types including 25 or 50 features. Measurements of model accuracy calculated by 5-fold cross validation technique included accuracy, true positive rate (sensitivity), true negative rate (specificity), and the area under the receiver operating curve (AUC).

## Results

### Resting-state EEG has nonlinear and perhaps chaotic structure in tinnitus and normal groups

We recorded resting-state EEG and reconstructed phase space of the underlying dynamical system for each channel of each participant using time-delay embedding of preprocessed EEG data (see methods). Nonlinearity of EEG data was confirmed with phase shuffled surrogate data [51]. Two measures of nonlinearity (lyapunov exponent and entropy) were calculated for the main and surrogate data. Compared to main data, surrogate data had lower lyapunov exponent (S1 Fig) and greater entropy (S2 Fig). Also, the distribution pattern of entropy and lyapunov exponent in EEG channels disappeared in surrogate data. This way, the null hypothesis of being generated by a stationary linear Gaussian process is rejected and existence of nonlinear structure is confirmed for both groups.

We computed the optimal lag and embedding dimension for each window separately. The embedding dimension was 4 in almost all channel-windows (96%), but lag values were variable for different channels and participants. We reconstructed phase space with dimension 4 and the most frequent value of lag in each subject-channel assuming that the underlying dynamical system of EEG windows only depends on the subject and the channel location. Optimizing the value of lag for each subject-channel separately allows for heterogeneous and at the same time comparative analysis of EEG data.

### In tinnitus group, lyapunov exponent and therefore the level of chaos is greater in frontal and left temporal areas

**Fig 1:**
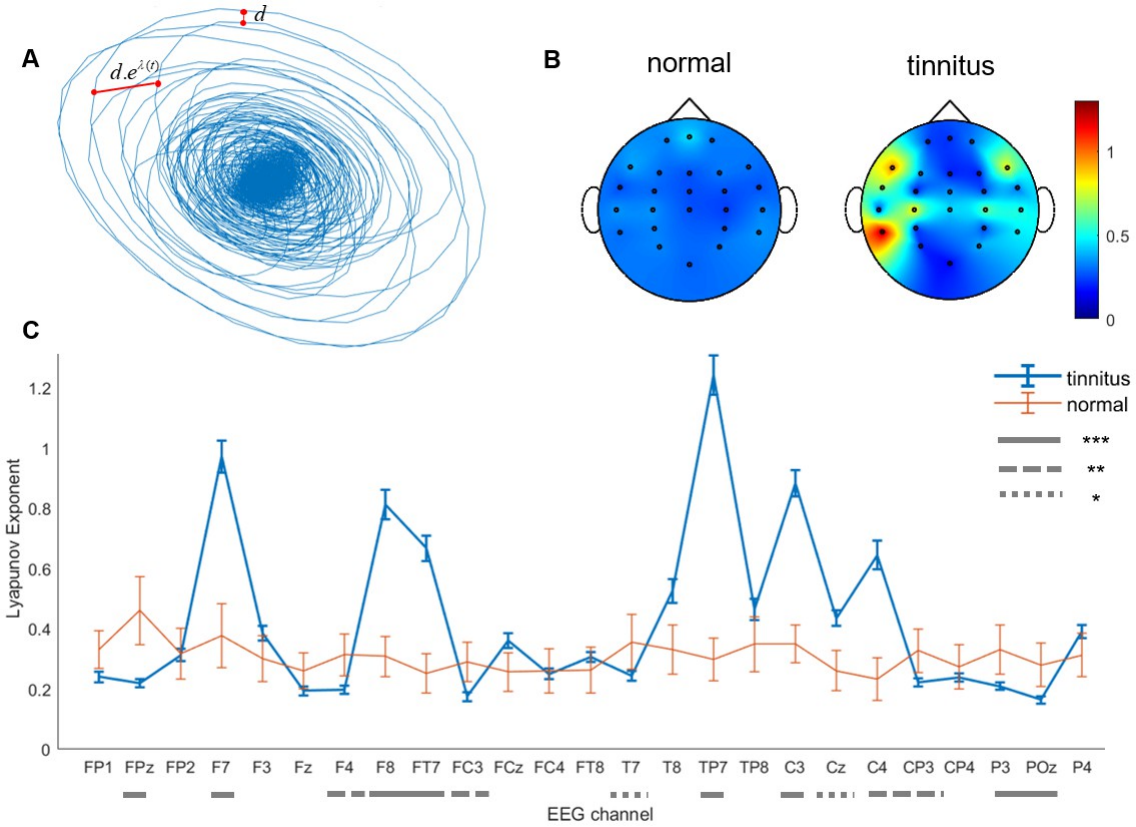
The balance of Lyapunov exponent is disturbed in subjects with tinnitus. (A) Example of a two-dimensional projection of the reconstructed phase space for 7 seconds of resting-state EEG for one participant with tinnitus. The schematic red lines demonstrate the concept of lyapunov exponent as the exponent of distance increase/decrease of nearby points on the attractor. (B) Topographic plots of mean lyapunov exponent in normal and patient groups. Temporal, frontal, and temporoparietal regions show significantly larger lyapunov exponents mostly in the left hemisphere in participants with tinnitus. (C) Lyapunov exponent in participants with tinnitus is greater than normal participants in F7, F8, FT7, FCz, T8, TP7, C3, Cz, and C4 channels corresponding to activities in vlPFC and OFC. In some frontal, parietal, and frontoparietal regions (FP1, FPz, F4, FC3, CP3, P3, POz corresponding to dlPFC, SMA, supramarginal and angular gyrus), lyapunov exponent in tinnitus groups is less than that of the control group. Error bars show the 95% confidence interval. See channel-wise p-values in S1 Table, *p < 0.05, **p < 0.01, *** p < 0.001.

Lyapunov exponent, used to quantify the level of chaos in a system is the rate of separation of infinitesimally close trajectories in the phase space [49] (Fig 1A) and is often calculated by quantifying the exponential increase or decrease of nearby point distances as the system evolves [30]. The balance of lyapunov exponent in normal subjects is disturbed in the tinnitus group. In subjects with tinnitus, lyapunov exponent is greater in temporal (mostly left) and frontal regions, specifically channels F7, F8, FT7, TP7, C3, C4(*p* < 0.001 for all), and lower in central regions, specifically channels FPz, F4, P3, POz (*p* < 0.001 for all), FC3 (*p* = 0.004), and CP3 (*p* = 0.008) (Fig 1B and 1C). Therefore, level of nonlinearity differs in the ventrolateral prefrontal cortex (vlPFC, BA44 and 45; F7, F8, FT7), middle temporal gyrus (MTG, BA21; TP7), primary somatosensory cortex (BA1; C3, C4), orbitofrontal cortex (OFC, BA10; FPz), right dlPFC (BA9; F4), left supplementary motor area (SMA, BA6; F4), supramarginal gyrus (BA40; CP3), angular gyrus (P3), cuneal cortex and precuneus (BA19; POz) in tinnitus patients. EEG channels are associated with Brodmann areas as suggested in [52]. Perhaps these regions actively participate in auditory perception. No significant difference was observed in parietal and prefrontal regions. Greater positive lyapunov exponents are associated with higher levels of chaos in a system. This suggests that tinnitus impacts the brain in widely distributed brain areas (see S1 Table for details on statistics).

### Tinnitus is correlated with higher entropy and therefore more complex dynamics in temporal and frontal areas

**Fig 2:**
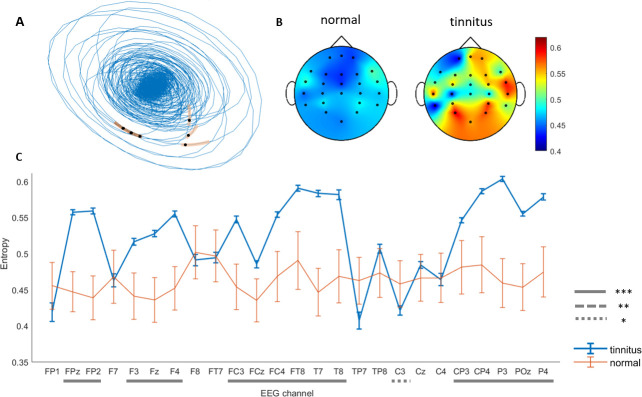
Tinnitus is correlated with higher levels of unpredictability in the brain. Entropy, schematically illustrated in (A) as the exponential growth of distinguishable orbits has a different pattern in tinnitus. (B) Topographic plot of entropy means illustrates higher entropy values in the right frontal and temporal regions in the tinnitus group. (C) Entropy is higher in tinnitus patients in channel FPz, FP2, F3, Fz, F4, FC3, FCz, FC4, FT8, T7, T8, CP3, CP4, P3, POz, and P4 corresponding to activities in OFC, dlPFC, MTG, STG, supramarginal and angular gyrus. But channel TP7 has a significantly lower entropy compared to the normal group. Error bars show the 95% confidence interval (See channel-wise p-values in S3 Table), *p < 0.05, **p < 0.01, *** p < 0.001.

Entropy is the exponential growth of distinguishable orbits [53] (Fig 2A) and is used as a measure of irregularity and unpredictability of nonlinear systems [42, 43]. Unlike lyapunov exponent which has a different focal pattern in tinnitus, entropy shows a regionally distributed distinct pattern in the patient group. In participants with tinnitus, entropy has greater values in channels FPz, FP2, F3, Fz, F4, FC3, FCz, FT8, T7, T8, CP3, CP4, P3, POz, and P4 (*p* < 0.001 for all) (Fig 2 and S3 Table). Thus, we observed enhanced entropy in OFC (BA10; FPz, FP2), dlPFC (BA9; F3, F4), SMA (BA6; Fz, FC3, FCz, FC4, FT8), middle and superior temporal gyrus (MTG, STG and Wernicke areas, BA21 and 22; T7, T8), supramarginal gyrus (BA40; CP3), angular gyrus (P3, P4, CP4), cuneal cortex and precuneus (BA19; POz) (Fig 2B and 2C). EEG channels are associated with Brodmann areas as suggested in [52].

### Nonlinear measures of dynamics are correlated between and across brain regions

**Fig 3:**
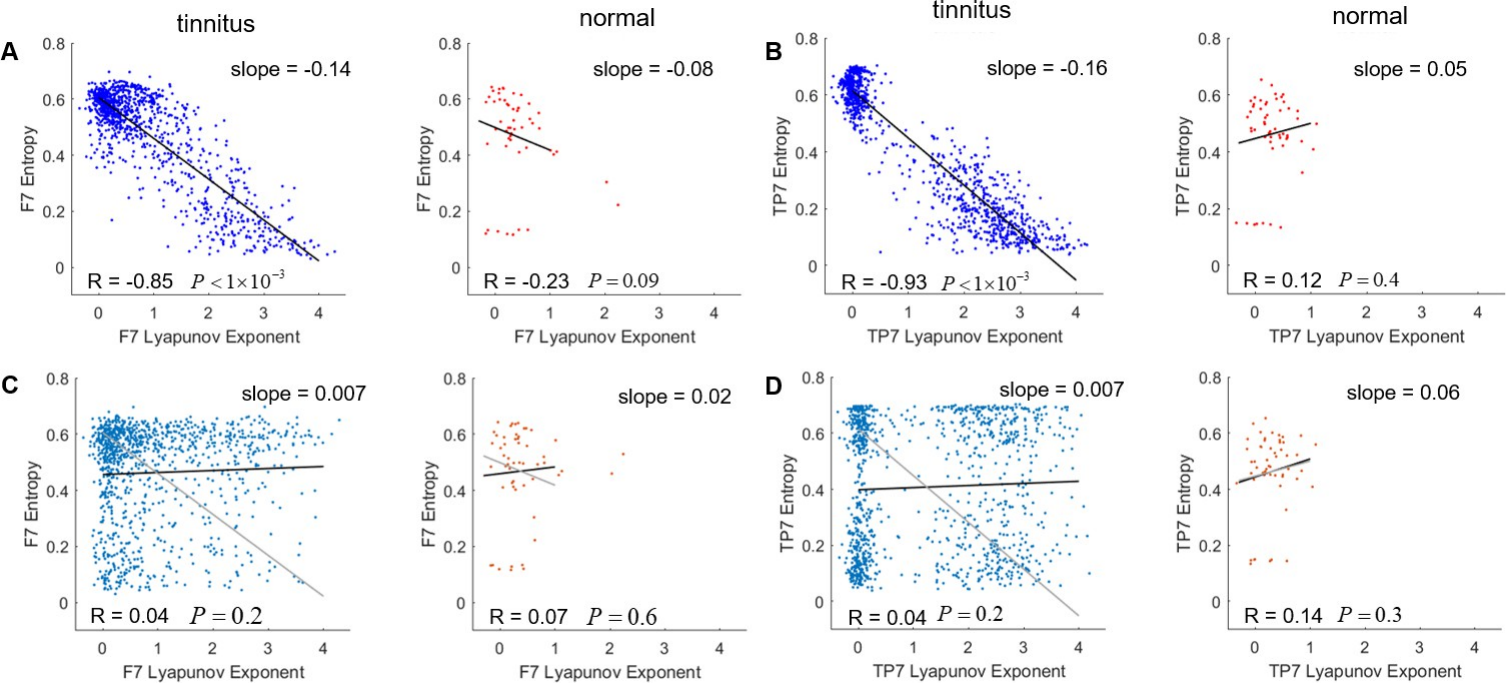
Entropy and lyapunov exponent are anticorrelated in some channels. Scatter plot of entropy vs. lyapunov exponent in channels F7 (A) and TP7 (B) with regression lines overlaid. Here R is the Pearson Correlation coefficient. These two measures of nonlinearity are highly anticorrelated in subjects who have tinnitus. To check significance of the observed anticorrelation, the Pearson Correlation coefficient was computed in surrogate data with shuffled trial labels. When trials are shuffled in channels F7 (C) and TP7 (D), the anticorrelation disappears. Regression lines of unshuffled data are plotted in gray.

During recording, subjects did not participate in any tasks however, measures of nonlinearity varied in different trials. If nonlinear measures did not represent the overall dynamics in the brain, then we would expect nonlinear features to be uncorrelated within and across different regions. However, in channel F7, near the left vlPFC, lyapunov exponent is anticorrelated with entropy only for tinnitus subjects (tinnitus, *R* = −0.87, *p* < 0.001; control, *R* = −0.23, *p* = 0.009) (Fig 3A). Also, in channel TP7 placed on the left temporal area significant anticorrelation between entropy and lyapunov exponent was observed only for the tinnitus group (tinnitus, *R* = −0.93, *p* < 0.001; control, *R* = 0.12, *p* = 0.4) (Fig 3B). Notably, the anticorrelation disappears with trial shuffling (Fig 3C and 3D). After shuffling trial labels, significant anticorrelation was observed neither in channel F7 (tinnitus, *R* = 0.04, *p* = 0.2; control, *R* = 0.07, *p* = 0.6) (Fig 3C), nor in channel TP7 (tinnitus, *R* = 0.04, *p* = 0.2; control, *R* = 0.14, *p* = 0.3) (Fig 3D).

**Fig 4:**
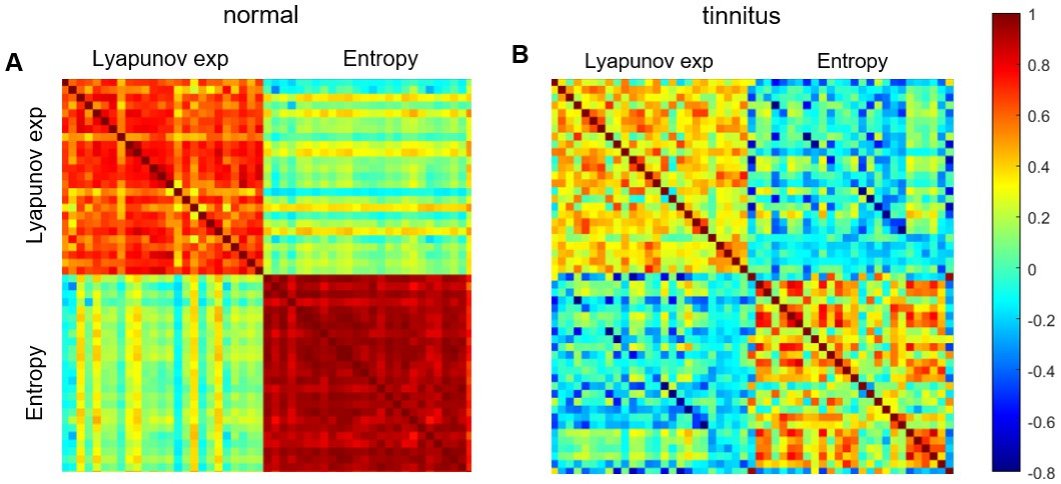
Cross-correlation map of nonlinear measures across EEG channels in normal (A) and tinnitus group (B). Each measure is correlated within EEG channels (red diagonal squares in A). Pearson correlation coefficient of entropy between different brain areas is lower in subjects with tinnitus. The same is true for lyapunov exponent. Correlation relations exist between nonlinear measures of different types. Entropy and lyapunov exponent become anticorrelated in tinnitus whereas no significant anticorrelation was observed in normal group. In tinnitus group few strong correlation relations (Pearson’s R > 0.7) was observed. In tinnitus group n = 2 channels pairs (Fig 5A and 5B) were strongly correlated whereas in control group n = 112 pairs including all channels except for F8, T8, TP8 (right frontotemporal areas) was observed. Entropy is correlated in all channels for control group (n = 412) but only n = 17 strongly correlated channel pairs exist in tinnitus group. Positively/negatively correlated areas in the tinnitus group are depicted in Fig 5.

Moreover, if tinnitus had no impact on global brain dynamics, then the correlation map of nonlinear measures would be similar for both groups. On the other hand, if tinnitus is associated with co-activation of auditory and non-auditory cortical areas, then the cross-correlation pattern of nonlinear measures in the tinnitus group must be different from that of the normal group. We found that such differentiation between groups is indeed the case (Fig 4) and observed patterns are different in the tinnitus group. Lyapunov exponent is correlated within (n = 299, p < 0.05) EEG channel pairs in the normal group whereas in the tinnitus group, less correlated channel pairs (n = 286, p < 0.05) were observed. Not only did the number of correlated channel pairs decrease, but the average correlation coefficient also falls in the tinnitus group (lyapunov exponent, mean R in tinnitus = 0.36, control = 0.64, t = 24.2, *p* < 0.001). The observed pattern is due to alterations in dynamics of brain areas as it is also observed in entropy. Entropy-correlated channel pairs decrease in number and mean correlation in subjects who have tinnitus (tinnitus: n = 285, mean R = 0.32; control: n = 300, mean R = 0.92; t = 38.7, *p* < 0.001). Plus, in normal subjects, entropy is strongly correlated in all recording sites (*p* < 0.001) whereas no such relation was observed in the tinnitus group (Fig 4 and S3 Fig).

### Tinnitus is associated with reorganization of paired EEG channels

**Fig 5:**
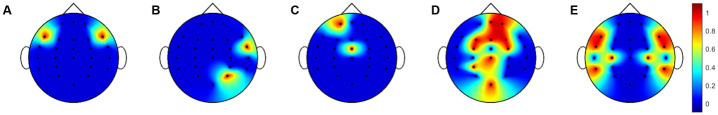
Strongly (anti)correlated channel sets in tinnitus group (|Pearson’s R| > 0.7) (A) Lyapunov exponent is positively correlated in F7 and F8 channels corresponding to left and right vlPFC. (B) Lyapunov exponent is positively correlated in channels FT8 and P4. Same is true for entropy in these channels. (C) Lyapunov exponent of FCz is anticorrelated with entropy of FP1 corresponding to anticorrelated activity in supplementary motor area (SMA) and orbitofrontal cortex (OFC). (D) Entropy is positively correlated within frontal and central region (channels FPz, Fz, F3, F4, FP2, FC3, FC4, CP3, Poz, C3). (E) Lyapunov exponent is anticorrelated with entropy in left and right temporal regions (channels F7, F8, FT7, T8, TP7, TP8, C3, C4). In each plot, color value is one for indicated channels and otherwise zero. For details on statistical significance see S5 Table.

Except for few remained correlated channel pairs, the whole correlation pattern between brain areas is lost in tinnitus (Fig 4). Focusing on strongly correlated pairs (|Pearson’s R| > 0.7), we demonstrate that lyapunov exponent is strongly correlated in n = 2 channel pairs in the tinnitus group (F7-F8 and FT8-P4) and in n = 112 channels pairs in the normal group. In subjects with tinnitus, F7 and F8 channels located at left and right vlPFC have positively correlated lyapunov exponent (*β* = 0.65, *R* = 0.70, *p* < 0.001) (Fig 5A), whereas in normal group lyapunov exponent correlation is weaker (*β* = 0.23, *R* = 0.37, *p* = 0.006). Besides, correlation of lyapunov exponent between FT8 and P4 channels (Fig 5B) in tinnitus (*β* = 0.94, *R* = 0.73, *p* < 0.001) is weaker than control group (*β* = 0.81, *R* = 0.84, *p* < 0.001). Entropy also remains highly correlated in these channels (tinnitus: *β* = 0.75, *R* = 0.71, *p* < 0.001; normal: *β* = 0.79, *R* = 0.91, *p* < 0.001). In tinnitus an entropy-correlated network is observed across FPz, Fz, F3, F4, FP2, FC3, FC4, CP3, Poz, C3 channels (Fig 5D) however, this network is significantly weaker in tinnitus compared to control group (tinnitus: Pearson’s R mean = 0.76; control: Pearson’s R mean = 0.92, *t* = 11.1, *p* < 0.001, See S5 Table for details).

In accordance with abnormal cortical activities, several brain areas appear to be strongly anticorrelated in tinnitus. Lyapunov exponent in FCz -located at supplementary motor cortex [52], is anticorrelated with entropy in FP1-located at orbitofrontal cortex (BA10) (*β* = −0.38, *R* = −0.72, *p* < 0.001) (Fig 5C). But in normal group no significant relation was observed (*p* = 0.24). In the tinnitus group, anticorrelation was observed between entropy and lyapunov exponent within (n = 22) and across (n = 353 pairs) EEG channels. These anticorrelated measures suggest and quantify a reorganization of cortical networks in tinnitus as previously reported [54].

Moreover, entropy-lyapunov exponent anticorrelation of individual channels is observed only in participants who have tinnitus (Fig 5E). (Tinnitus: Pearson’s R mean = -0.83, See S5 Table for details.) Appearance of strong anticorrelations in temporal, frontal, and parietal regions in the tinnitus group suggests that tinnitus is associated with abnormal oscillatory dynamics and increased synchronous activities in a wide range of brain areas. The (anti)correlated channel sets (Fig 5) correspond to brain areas involved in phantom auditory perception reported previously in various neural mechanisms for tinnitus (See Discussion).

### Nonlinear measures from chaos theory gives a classification of subjects with and without tinnitus

**Fig 6:**
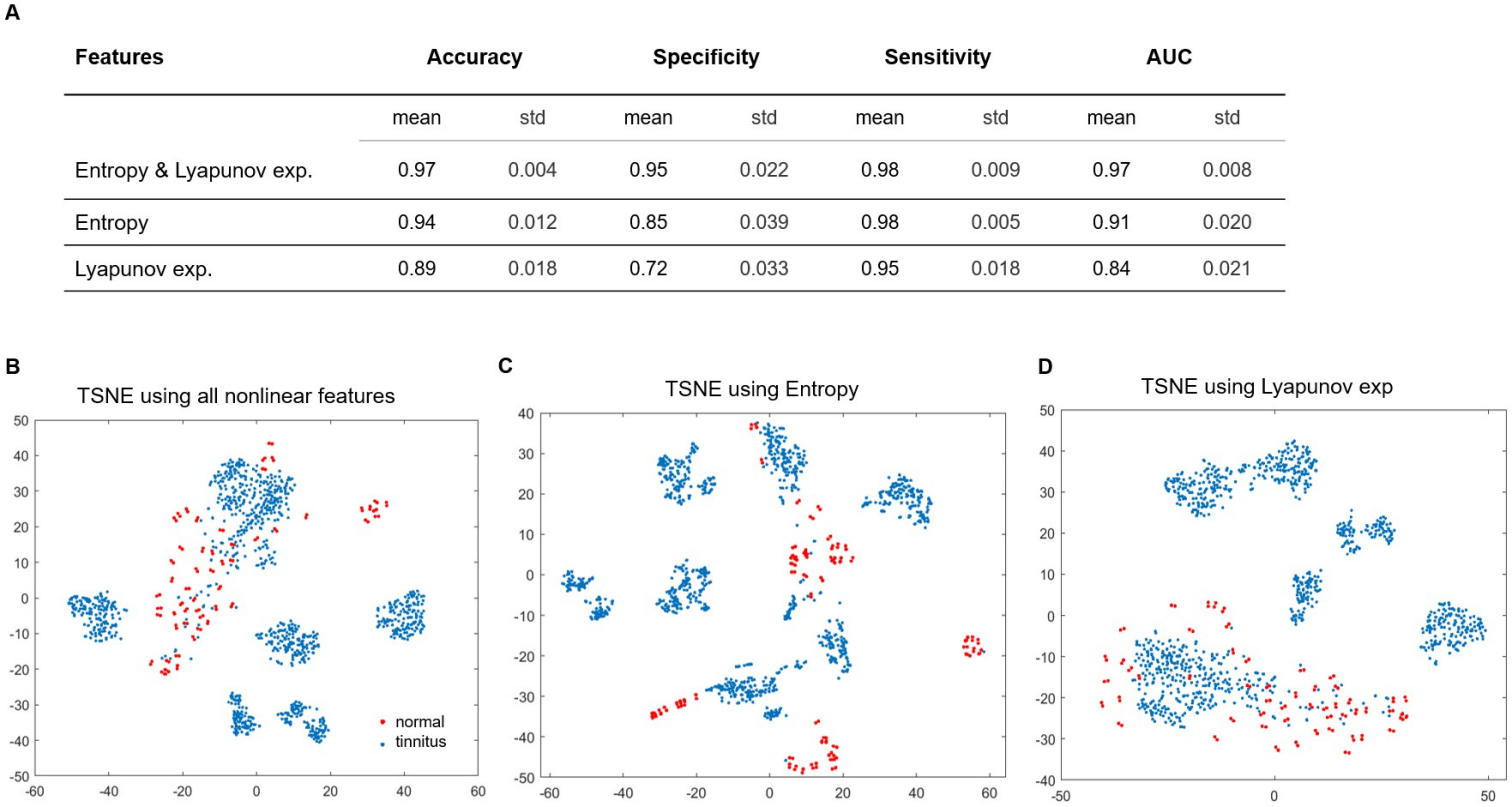
Tinnitus and normal participants can be separated using linear SVM classifiers on nonlinear dynamical features. (A) Mean and standard deviation of Accuracy, Specificity, Sensitivity, and AUC of linear SVM classifiers for a specified set of features of tinnitus (w = 1) and normal (w = 7) participants using 5-fold cross validation. The features column indicates the set of nonlinear measures used as features in the training stage. Tinnitus and normal groups are differentiated in the TSNE plot of all nonlinear features (B), entropy (C), and Lyapunov exponent (D) of all channels. Also, the visible clusters in the tinnitus group (blue dots) may explain tinnitus heterogeneity.

Finally, training a linear SVM based on lyapunov exponent and entropy of all channels as features of each trial, we obtained a classifier with an accuracy of 0.97 (std = 0.004) specificity of 0.95 (std = 0.022), and sensitivity of 0.98 (std = 0.009) through from 5-fold cross validation (see methods for details). Using only one nonlinear measure as training feature, classifiers of less but acceptable power were attained (Fig 6A). In order to understand this high-dimensional data we projected nonlinear measures of all trials into two-dimensional space using t-distributed stochastic neighbor embedding (t-SNE). As observed in t-SNE plots (Fig 6), several clusters appear in the tinnitus group that may correspond to various phenotypes. Attribution of clusters to tinnitus phenotypes needs further investigation of large datasets including heterogeneous symptoms of tinnitus in participants.

## Discussion

About 85% of chronic tinnitus sufferers perceive the tinnitus sound constantly [55]. Therefore, resting-state recordings seem appropriate to reflect neural correlates of tinnitus. In this study, we demonstrated that nonlinear measures, namely lyapunov exponent and entropy may be utilized as metrics to quantify complexity and irregularity of brain nonlinear dynamics in resting-state EEG recordings of tinnitus. Since subjects with concurrent and complicating symptoms and diseases were excluded from potential participants, all observed effects between the study groups is assumed to be due to tinnitus.

Studies of tinnitus animal models have shown that peripheral deafferentation results in increased spontaneous firing rate and bursting of neurons in the auditory pathway [11, 56]. This increase in spontaneous neuronal activity is explained by excitation-inhibition imbalance [57]; a decrease in neuronal inhibition and an increase in excitation due to alterations in GABAergic, glycinergic, and glutamatergic neurotransmission [58]. This imbalance is also observed when comparing nonlinear measures as well as cross correlation patterns of cortical areas of control group with subjects who have tinnitus.

Our results provide evidence for existence of abnormal signals across a large anatomical area including auditory and non-auditory regions [59, 60]. The imbalance of entropy and lyapunov exponent supports occurrence of a shift in the balance of excitation and inhibition in cortical networks [57] as well as reduced GABA concentration in auditory cortex of subjects with tinnitus [61]. We demonstrate that in tinnitus group, lyapunov exponent is higher in left temporal and frontal regions (Fig 1) supporting the fact that tinnitus is associated with excessive activation in left auditory and anterior cingulate cortex [62]. This increase in temporal and frontal regions is also consistent with the synchronized firing of neurons in primary and secondary auditory cortex [9, 63], fusiform cells [64, 65], and increased connectivity within auditory cortex [23]. Besides, the decrease in lyapunov exponent in FC3, FPz, and F4 may be due to pathological loss of complexity or limit cycle dynamics in tinnitus.

Several mechanisms have been proposed as the neural basis of tinnitus including permanent alterations in the ongoing oscillatory dynamics at higher layers of the auditory hierarchical stream [25]. One proposed mechanism for tinnitus suggests that regulations of neuronal excitability in central auditory areas is in response to reduced auditory cochlear input. This compensatory reaction also amplifies “neural noise” which is perceived as tinnitus [9]. In other words, auditory deafferentation limits the amount of information and leads to increased uncertainty in the brain and tinnitus is the brain attempt to fill in the missing information [62]. Accordingly, we have demonstrated that entropy which is a measure of uncertainty, has higher values in OFC, dlPFC, SMA, and MTG areas in tinnitus subjects, consistent with abnormal activities observed in inferior temporal gyrus (ITG) and MTG [62]. This could be due to inconsistency in parallel auditory processes and therefore higher levels of ambiguity and uncertainty in the brain. We found that nonlinear measures also differentiate tinnitus confirming abnormal oscillatory brain dynamics in tinnitus patients [25].

Tinnitus pathology involves auditory and non-auditory brain areas [66, 67]. While tinnitus perception is related to abnormal neural activity in the auditory pathway, tinnitus distress is associated with increased co-activation of frontal, limbic, memory and autonomic brain areas [66]. In this regard, our correlation analysis reveals (anti)correlated networks across different parts of the brain. It is also proposed that as tinnitus becomes more severe, spontaneous fluctuations of the precuneus begin to switch from their usual patterns (leading to strong correlations with other regions in the default mode network) to more closely resemble the patterns seen in the dorsal attention network [68]. Consistently, we found that FCz-FP1 anticorrelation is strengthened in tinnitus and FT8-P4 correlation is weakened simultaneously.

The observed entropy-lyapunov exponent correlated network including F3, F4, Fz, and POz channels may correspond to increased synchronized activity in ACC observed in tinnitus patients with hearing loss [62]. Right and left temporal areas show entropy-lyapunov exponent anticorrelation in tinnitus (Fig 5E). Knowing that entropy is a measure of unpredictability and that higher lyapunov exponent is associated with higher levels of chaos in a system, we conclude that unpredictable chaotic dynamics of temporal areas in tinnitus is generated by a common cause. This common cause could be habituation to tinnitus sound [24] or network alterations in the auditory stream [67]. Interestingly, this anticorrelation in tinnitus leads to higher lyapunov exponent in left temporal cortex (Fig 1) and higher entropy in right temporal areas (Fig 2). This is consistent with reports indicating different roles of right and left brain regions [13].

On the other hand, anticorrelation between channels FCz and FP1 in tinnitus could be due to decreased effective connectivity between SMA and OFC corresponding to deficits in working memory and attention in tinnitus patients [69–71].

To summarize, nonlinear analysis of brain dynamics in resting-state EEG recordings gives rise to indicators of tinnitus in individual channels and across cortical areas. These indicators advocate a variety of tinnitus mechanisms and are consistent with the results of many tinnitus studies. Heterogeneity and independence from functional frequency bands makes the proposed method suitable for screening tinnitus patients. Even before emergence of tinnitus symptoms, perhaps at early stages, these indicators could potentially be used to indicate the risk of developing tinnitus in the future. Knowledge of tinnitus risk can be beneficial in preventing severe symptoms as well as finding novel approaches for tinnitus treatment. If confirmed through usage of large datasets, this method could potentially be employed in clinical diagnosis of the heterogeneous nature of tinnitus.

## Supporting information

S1 Data Anonymized data. (ZIP)

S1 Fig EEG data has nonlinear structure in normal and patient groups as reflected in lyapunov exponent. In phase shuffled surrogate data, the channel-wise pattern of lyapunov exponent is lost. In participants with tinnitus, lyapunov exponent (blue line) is greater than phase shuffled surrogates (blue dashed-line) confirming the nonlinear and perhaps chaotic dynamics of tinnitus EEG recordings. But lyapunov exponents in the normal group (red line) are near the same values for phase shuffled surrogates (red dashed-line) indicating the weak nonlinearity of brain dynamics in healthy participants during rest. Error bars show the 95% confidence interval. See channel wise p-values in S2 Table. *p < 0.05, **p < 0.01, *** p < 0.001.
(TIF)

S2 Fig EEG data has nonlinear structure in normal and patient groups as reflected in entropy. The channel-wise pattern in entropy is lost in surrogate data. Entropy of surrogate data is almost similar and for all channels (except for FP1) in each group. In both tinnitus and normal groups, Entropy (solid lines) is less than phase shuffled surrogates (dashed-lines). This confirms that although brain dynamics is complex, it has a meaningful structure. Error bars show the 95% confidence interval. See channel wise p-values in S4 Table. *p < 0.05, **p < 0.01, *** p < 0.001.
(TIF)

S3 Fig Significance of cross-correlation map of nonlinear measures across EEG channels. Color-coded display of p-values for Pearson Correlation coefficient in different channel-metrics in normal (A) and tinnitus group (B).
(TIF)

S1 Table Statistical significance of Lyapunov exponent between tinnitus and normal groups using two-sided two-sampled t-tests. (PDF)

S2 Table Nonlinear structure of resting-state EEG recordings is confirmed using phase shuffled surrogate data. Details of statistical significance of lyapunov exponent difference in normal and tinnitus groups using two-sided two-sampled t-tests.
(PDF)

S3 Table Statistical significance of entropy difference between tinnitus and normal groups using two-sided two-sampled t-tests. (PDF)

S4 Table Nonlinear structure of resting-state EEG recordings is confirmed using phase shuffled surrogate data. Details of statistical significance of entropy difference in normal and tinnitus groups using two-sided two-sampled t-tests.
(PDF)

S5 Table Statistical significance of strongly (anti)correlated channel sets in tinnitus group (|Pearson’s R| > 0.7) R is Pearson’s correlation coefficient and beta is the slope of the regression line. Sign shows the sign of Pearson’s R difference between tinnitus and normal group and is not specified for non-significant values.
(PDF)
