# Supplementary figures and images for: Heterogeneous correlate and potential diagnostic biomarker of tinnitus based on nonlinear dynamics of resting-state EEG recordings
Source: PLoS One. 2024 Jan 2;19(1):e0290563. doi: 10.1371/journal.pone.0290563 (PMC10760901; doi:10.1371/journal.pone.0290563)

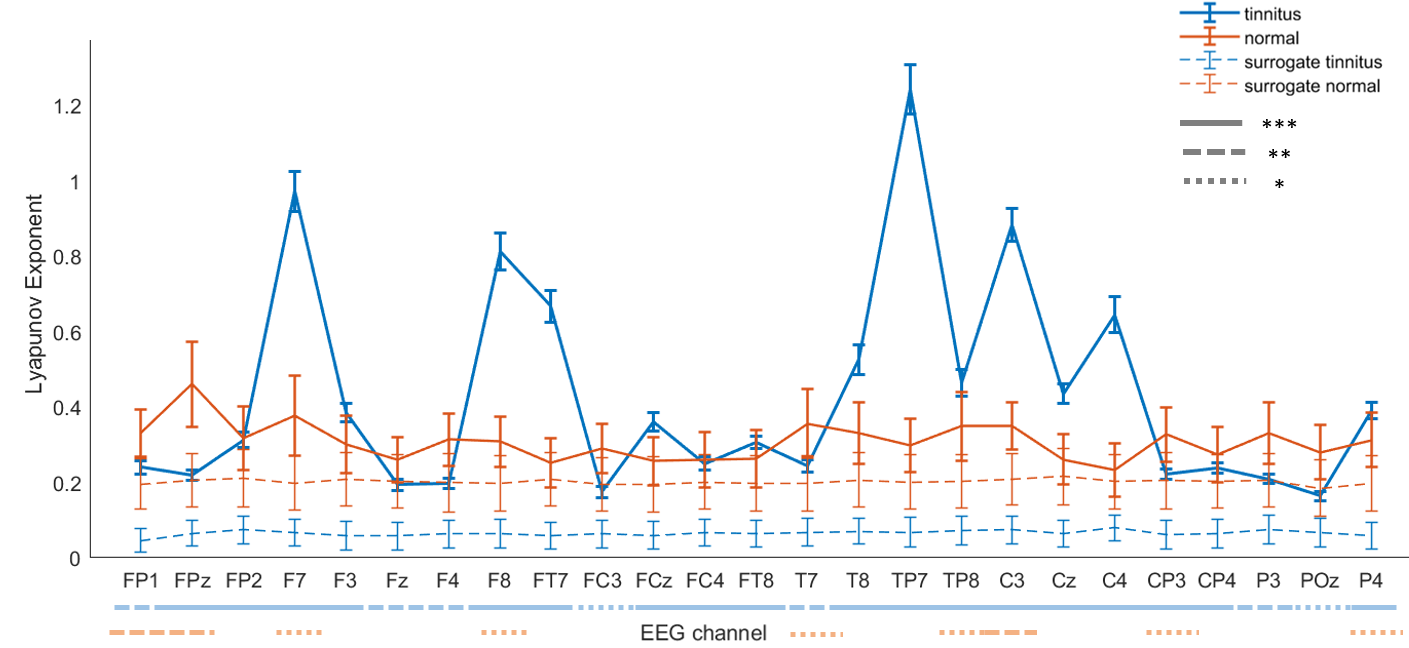

Supplement: S1 Fig — In phase shuffled surrogate data, the channel-wise pattern of lyapunov exponent is lost. In participants with tinnitus, lyapunov exponent (blue line) is greater than phase shuffled surrogates (blue dashed-line) confirming the nonlinear and perhaps chaotic dynamics of tinnitus EEG recordings. But lyapunov exponents in the normal group (red line) are near the same values for phase shuffled surrogates (red dashed-line) indicating the weak nonlinearity of brain dynamics in healthy participants during rest. Error bars show the 95% confidence interval. See channel wise p-values in S2 Table. *p < 0.05, **p < 0.01, *** p < 0.001. (TIF) [file pone.0290563.s002.tif]

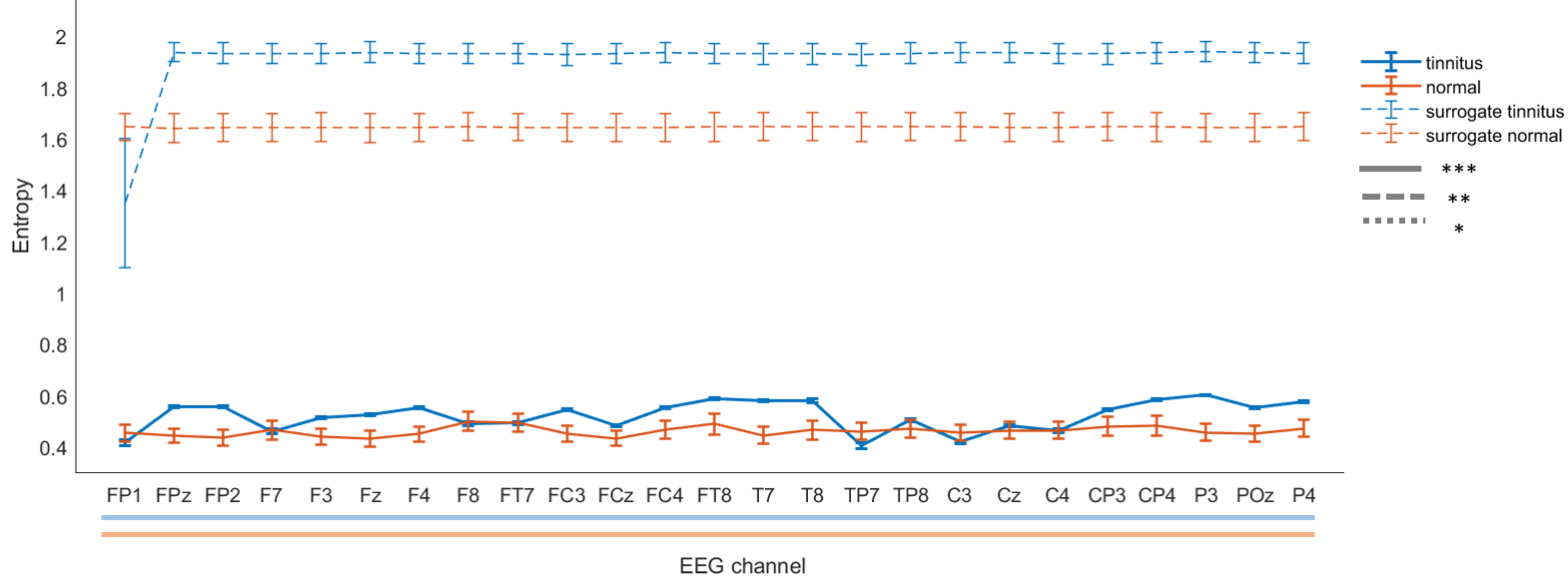

Supplement: S2 Fig — The channel-wise pattern in entropy is lost in surrogate data. Entropy of surrogate data is almost similar and for all channels (except for FP1) in each group. In both tinnitus and normal groups, Entropy (solid lines) is less than phase shuffled surrogates (dashed-lines). This confirms that although brain dynamics is complex, it has a meaningful structure. Error bars show the 95% confidence interval. See channel wise p-values in S4 Table. *p < 0.05, **p < 0.01, *** p < 0.001. (TIF) [file pone.0290563.s003.tif]

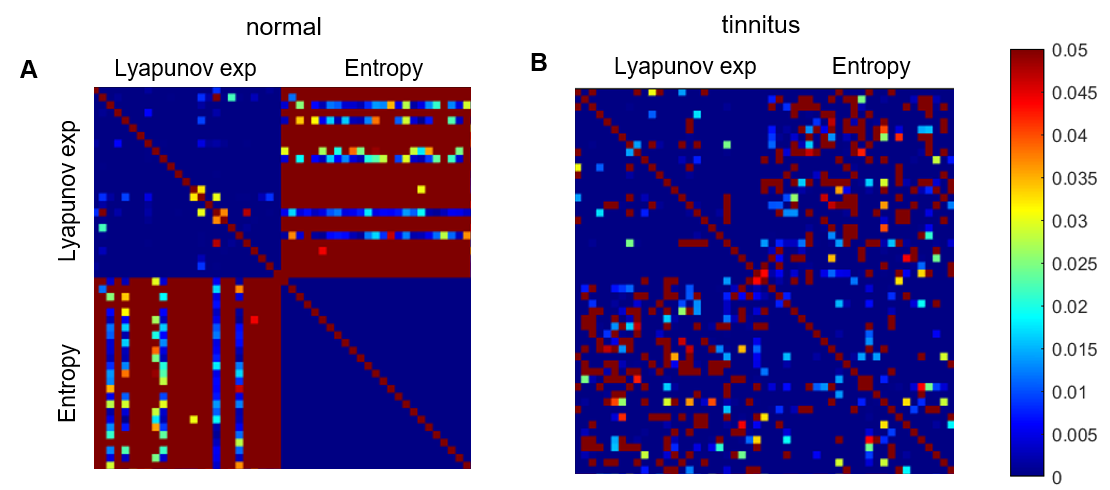

Supplement: S3 Fig — Color-coded display of p-values for Pearson Correlation coefficient in different channel-metrics in normal (A) and tinnitus group (B). (TIF) [file pone.0290563.s004.tif]
