# Supplementary material for: Heterogeneous correlate and potential diagnostic biomarker of tinnitus based on nonlinear dynamics of resting-state EEG recordings
Source: PLoS One. 2024 Jan 2;19(1):e0290563. doi: 10.1371/journal.pone.0290563 (PMC10760901; doi:10.1371/journal.pone.0290563)
Supplement: S1 Table — (PDF) [file pone.0290563.s005.pdf]

Channel name	normal mean	tinnitus mean	t-statistics	p-value	significance level
FP1	0.33	0.24	1.84	6.65E-02	
FPz	0.46	0.22	6.12	1.34E-09	***
FP2	0.32	0.31	0.07	9.41E-01	
F7	0.38	0.97	-4.14	3.71E-05	***
F3	0.30	0.39	-1.21	2.26E-01	
Fz	0.26	0.19	1.68	9.38E-02	
F4	0.31	0.20	3.26	1.14E-03	**
F8	0.31	0.81	-3.79	1.62E-04	***
FT7	0.25	0.67	-3.62	3.04E-04	***
FC3	0.29	0.17	2.89	3.89E-03	**
FCz	0.26	0.36	-1.58	1.15E-01	
FC4	0.26	0.25	0.23	8.20E-01	
FT8	0.26	0.31	-1.00	3.16E-01	
T7	0.36	0.24	2.44	1.50E-02	*
T8	0.33	0.53	-1.80	7.18E-02	
TP7	0.30	1.24	-5.33	1.22E-07	***
TP8	0.35	0.46	-1.22	2.23E-01	
C3	0.35	0.88	-4.48	8.36E-06	***
Cz	0.26	0.44	-2.51	1.22E-02	*
C4	0.23	0.65	-3.23	1.28E-03	**
CP3	0.33	0.22	2.67	7.69E-03	**
CP4	0.27	0.24	0.90	3.68E-01	
P3	0.33	0.21	3.43	6.20E-04	***
POz	0.28	0.16	3.54	4.19E-04	***
P4	0.31	0.39	-1.35	1.78E-01	
