# Supplementary material for: Heterogeneous correlate and potential diagnostic biomarker of tinnitus based on nonlinear dynamics of resting-state EEG recordings
Source: PLoS One. 2024 Jan 2;19(1):e0290563. doi: 10.1371/journal.pone.0290563 (PMC10760901; doi:10.1371/journal.pone.0290563)
Supplement: S2 Table — Details of statistical significance of lyapunov exponent difference in normal and tinnitus groups using two-sided two-sampled t-tests. (PDF) [file pone.0290563.s006.pdf]

	NORMAL GROUP					TINNITUS GROUP				
CHANNEL	mean	Surrogate mean	t-statistics	p-value	significance level	mean	surrogate mean	t-statistics	p-value	significance level
FP1	0.33	0.18	2.72	7.95E-03	**	0.24	0.05	3.23	1.29E-03	**
FPZ	0.46	0.20	3.01	3.39E-03	**	0.22	0.07	3.45	5.74E-04	***
FP2	0.32	0.20	1.62	1.09E-01		0.31	0.07	3.51	4.67E-04	***
F7	0.38	0.19	2.18	3.20E-02	*	0.97	0.07	5.14	3.28E-07	***
F3	0.30	0.20	1.62	1.08E-01		0.39	0.06	3.87	1.14E-04	***
FZ	0.26	0.19	1.28	2.03E-01		0.19	0.06	2.83	4.67E-03	**
F4	0.31	0.19	1.98	5.03E-02		0.20	0.06	3.15	1.67E-03	**
F8	0.31	0.19	2.01	4.77E-02	*	0.81	0.06	4.56	5.60E-06	***
FT7	0.25	0.20	0.91	3.65E-01		0.67	0.06	4.32	1.67E-05	***
FC3	0.29	0.19	1.78	7.90E-02		0.17	0.06	2.31	2.09E-02	*
FCZ	0.26	0.19	1.24	2.17E-01		0.36	0.06	3.71	2.16E-04	***
FC4	0.26	0.20	1.01	3.18E-01		0.25	0.07	3.14	1.73E-03	***
FT8	0.26	0.19	1.14	2.57E-01		0.31	0.06	4.48	8.14E-06	***
T7	0.36	0.19	2.26	2.63E-02	*	0.24	0.07	3.22	1.34E-03	**
T8	0.33	0.20	1.97	5.17E-02		0.53	0.07	3.42	6.49E-04	***
TP7	0.30	0.19	1.77	8.07E-02		1.24	0.07	5.39	8.73E-08	***
TP8	0.35	0.20	2.09	3.95E-02	*	0.46	0.07	3.39	7.14E-04	***
C3	0.35	0.20	2.71	8.16E-03	**	0.88	0.07	5.53	3.95E-08	***
CZ	0.26	0.21	0.96	3.38E-01		0.44	0.06	4.39	1.24E-05	***
C4	0.23	0.20	0.63	5.33E-01		0.65	0.08	3.61	3.21E-04	***
CP3	0.33	0.20	2.10	3.89E-02	*	0.22	0.06	3.41	6.71E-04	***
CP4	0.27	0.19	1.30	1.96E-01		0.24	0.06	3.82	1.40E-04	***
P3	0.33	0.20	1.98	5.06E-02		0.21	0.07	3.21	1.35E-03	**
POZ	0.28	0.18	1.70	9.37E-02		0.16	0.07	2.50	1.26E-02	*
P4	0.31	0.19	1.99	4.99E-02	*	0.39	0.06	4.75	2.32E-06	***
