# Supplementary material for: Heterogeneous correlate and potential diagnostic biomarker of tinnitus based on nonlinear dynamics of resting-state EEG recordings
Source: PLoS One. 2024 Jan 2;19(1):e0290563. doi: 10.1371/journal.pone.0290563 (PMC10760901; doi:10.1371/journal.pone.0290563)
Supplement: S3 Table — (PDF) [file pone.0290563.s007.pdf]

channel	normal mean	tinnitus mean	t-statistics	p-value	significance level
FP1	0.46	0.42	1.05	3.0E-01	
FPz	0.45	0.56	-10.51	1.0E-24	***
FP2	0.44	0.56	-10.12	4.5E-23	***
F7	0.47	0.46	0.20	8.4E-01	
F3	0.44	0.52	-5.65	2.1E-08	***
Fz	0.44	0.53	-7.57	8.0E-14	***
F4	0.45	0.56	-9.02	8.4E-19	***
F8	0.50	0.49	0.48	6.3E-01	
FT7	0.50	0.49	0.11	9.1E-01	
FC3	0.45	0.55	-6.86	1.1E-11	***
FCz	0.44	0.49	-3.49	5.1E-04	***
FC4	0.47	0.55	-7.59	6.8E-14	***
FT8	0.49	0.59	-7.86	8.8E-15	***
T7	0.45	0.58	-11.24	7.9E-28	***
T8	0.47	0.58	-5.88	5.4E-09	***
TP7	0.46	0.41	1.73	8.4E-02	
TP8	0.47	0.51	-1.91	5.6E-02	
C3	0.46	0.42	2.00	4.5E-02	*
Cz	0.47	0.48	-1.30	1.9E-01	
C4	0.47	0.46	0.10	9.2E-01	
CP3	0.48	0.55	-6.05	1.9E-09	***
CP4	0.48	0.59	-9.22	1.4E-19	***
P3	0.46	0.60	-13.25	2.6E-37	***
POz	0.45	0.56	-10.64	3.2E-25	***
P4	0.47	0.58	-8.00	3.2E-15	***
