# Supplementary material for: Heterogeneous correlate and potential diagnostic biomarker of tinnitus based on nonlinear dynamics of resting-state EEG recordings
Source: PLoS One. 2024 Jan 2;19(1):e0290563. doi: 10.1371/journal.pone.0290563 (PMC10760901; doi:10.1371/journal.pone.0290563)
Supplement: S4 Table — Details of statistical significance of entropy difference in normal and tinnitus groups using two-sided two-sampled t-tests. (PDF) [file pone.0290563.s008.pdf]

	NORMAL GROUP					TINNITUS GROUP				
CHANNEL	mean	Surrogate mean	t-statistics	p-value	significance level	mean	surrogate mean	t-statistics	p-value	significance level
FP1	0.46	1.65	-35.78	1.99E-53	***	0.42	1.35	-18.55	6.12E-67	**
FPZ	0.45	1.64	-38.22	9.55E-56	***	0.56	1.94	-108.53	<1.0E-99	***
FP2	0.44	1.64	-37.11	1.06E-54	***	0.56	1.93	-95.35	<1.0E-99	***
F7	0.47	1.64	-32.94	1.53E-50	***	0.46	1.93	-48.34	<1.0E-99	***
F3	0.44	1.65	-35.86	1.69E-53	***	0.52	1.93	-87.95	<1.0E-99	***
FZ	0.44	1.64	-36.79	2.14E-54	***	0.53	1.94	-95.98	<1.0E-99	**
F4	0.45	1.65	-36.90	1.66E-54	***	0.56	1.93	-99.83	<1.0E-99	**
F8	0.50	1.65	-31.84	2.25E-49	***	0.49	1.93	-53.29	<1.0E-99	***
FT7	0.50	1.65	-32.46	4.96E-50	***	0.49	1.93	-59.07	<1.0E-99	***
FC3	0.45	1.64	-35.89	1.58E-53	***	0.55	1.93	-83.39	<1.0E-99	*
FCZ	0.44	1.64	-37.62	3.50E-55	***	0.49	1.93	-82.92	<1.0E-99	***
FC4	0.47	1.65	-33.90	1.54E-51	***	0.55	1.94	-103.31	<1.0E-99	***
FT8	0.49	1.65	-30.12	1.82E-47	***	0.59	1.93	-90.50	<1.0E-99	***
T7	0.45	1.65	-35.35	5.37E-53	***	0.58	1.93	-92.34	<1.0E-99	**
T8	0.33	0.20	1.97	5.17E-02		0.53	0.07	3.42	6.49E-04	***
TP7	0.30	0.19	1.77	8.07E-02		1.24	0.07	5.39	8.73E-08	***
TP8	0.35	0.20	2.09	3.95E-02	*	0.46	0.07	3.39	7.14E-04	***
C3	0.35	0.20	2.71	8.16E-03	**	0.88	0.07	5.53	3.95E-08	***
CZ	0.26	0.21	0.96	3.38E-01		0.44	0.06	4.39	1.24E-05	***
C4	0.23	0.20	0.63	5.33E-01		0.65	0.08	3.61	3.21E-04	***
CP3	0.33	0.20	2.10	3.89E-02	*	0.22	0.06	3.41	6.71E-04	***
CP4	0.27	0.19	1.30	1.96E-01		0.24	0.06	3.82	1.40E-04	***
P3	0.33	0.20	1.98	5.06E-02		0.21	0.07	3.21	1.35E-03	**
POZ	0.28	0.18	1.70	9.37E-02		0.16	0.07	2.50	1.26E-02	*
P4	0.31	0.19	1.99	4.99E-02	*	0.39	0.06	4.75	2.32E-06	***
