# Supplementary material for: Heterogeneous correlate and potential diagnostic biomarker of tinnitus based on nonlinear dynamics of resting-state EEG recordings
Source: PLoS One. 2024 Jan 2;19(1):e0290563. doi: 10.1371/journal.pone.0290563 (PMC10760901; doi:10.1371/journal.pone.0290563)
Supplement: S5 Table — Sign shows the sign of Pearson’s R difference between tinnitus and normal group and is not specified for non-significant values. (PDF) [file pone.0290563.s009.pdf]

Channel-metric		Normal group			Tinnitus group			Sign
		beta	R	p-value	beta	R	p-value	($R_{tinnitus} - R_{normal}$)
FT8 Lyapunov	P4 Lyapunov	0.81	0.84	3.90E-15	0.94	0.73	2.91E-178	-
F7 Lyapunov	F8 Lyapunov	0.23	0.37	6.00E-03	0.65	0.70	3.91E-158	+
FPz Entropy	FP2 Entropy	1.02	0.94	4.55E-25	0.80	0.70	1.33E-157	-
FPz Entropy	Fz Entropy	1.02	0.91	1.37E-21	0.95	0.82	1.06E-253	-
FPz Entropy	F4 Entropy	0.99	0.91	1.66E-21	0.83	0.76	2.48E-196	-
FP2 Entropy	F4 Entropy	0.98	0.98	6.30E-37	0.70	0.73	1.31E-175	-
Fz Entropy	F4 Entropy	0.94	0.97	8.29E-33	0.76	0.82	1.21E-253	-
FPz Entropy	FC3 Entropy	0.99	0.87	1.19E-17	1.00	0.75	6.17E-193	-
F3 Entropy	FC3 Entropy	0.94	0.96	5.36E-30	0.79	0.77	3.78E-206	-
Fz Entropy	FC3 Entropy	0.98	0.97	1.82E-34	0.85	0.75	2.10E-190	-
Fz Entropy	FC4 Entropy	1.05	0.95	4.22E-27	0.65	0.72	2.78E-172	-
F4 Entropy	FC4 Entropy	1.12	0.98	1.04E-38	0.80	0.83	7.94E-272	-
F3 Entropy	Cz Entropy	0.88	0.87	1.19E-17	0.74	0.71	7.28E-164	-
FPz Entropy	CP3 Entropy	1.21	0.91	2.18E-21	0.73	0.75	3.81E-191	-
Fz Entropy	CP3 Entropy	1.03	0.86	8.84E-17	0.64	0.76	1.83E-199	-
FC3 Entropy	CP3 Entropy	1.00	0.85	6.75E-16	0.56	0.76	7.58E-203	-
FPz Entropy	POz Entropy	1.00	0.86	2.67E-16	0.63	0.73	4.63E-173	-
Fz Entropy	POz Entropy	0.99	0.95	1.01E-27	0.57	0.77	6.82E-205	-
CP3 Entropy	POz Entropy	0.76	0.87	2.85E-17	0.76	0.85	1.03E-292	-
FT8 Entropy	P4 Entropy	0.79	0.91	6.71E-21	0.75	0.71	2.83E-162	-
T8 Lyapunov	T8 Entropy	-0.06	-0.14	3.29E-01	-0.15	-0.86	1.64E-304	
TP7 Lyapunov	TP7 Entropy	0.05	0.12	4.00E-01	-0.17	-0.93	0.00E+00	
TP8 Lyapunov	TP8 Entropy	0.13	0.34	1.36E-02	-0.13	-0.74	8.17E-186	-
C3 Lyapunov	C3 Entropy	0.01	0.02	8.64E-01	-0.12	-0.78	5.64E-216	
C4 Lyapunov	C4 Entropy	0.18	0.38	5.10E-03	-0.15	-0.85	1.64E-288	-
F8 Lyapunov	F8 Entropy	-0.01	-0.02	8.89E-01	-0.13	-0.83	2.53E-266	
FT7 Lyapunov	FT7 Entropy	0.14	0.25	7.16E-02	-0.13	-0.76	4.21E-199	
F7 Lyapunov	F7 Entropy	-0.08	-0.23	9.13E-02	-0.15	-0.85	7.47E-301	
FCz Lyapunov	FP1 Entropy	0.08	0.16	2.41E-01	-0.38	-0.72	8.00E-172	
